# Efficacy of a Web‐Based Tool in Reducing Burnout Among Behavioral Health Clinicians: Results From the PTSD Clinicians Exchange

**DOI:** 10.1176/appi.prcp.20190004

**Published:** 2020-09-09

**Authors:** Kristina Clarke‐Walper, Elizabeth A. Penix, Felicia Trachtenberg, Erica Simon, Julia Coleman, Ashley Magnavita, Kile Ortigo, Samantha Regala, Lisa Marceau, Josef I. Ruzek, Raymond C. Rosen, Joshua E. Wilk

**Affiliations:** ^1^ Center for Military Psychiatry and Neurosciences Department of Military Psychiatry Walter Reed Army Institute of Research Silver Spring Maryland; ^2^ New England Research Institutes Watertown Massachusetts; ^3^ National Center for PTSD Dissemination and Training Division U.S. Department of Veterans Affairs Palo Alto Health Care System Menlo Park California; ^4^ Palo Alto Veterans Institute for Research Palo Alto California; ^5^ Department of Psychiatry and Behavioral Sciences Stanford University Stanford California

**Keywords:** Military psychiatry, Posttraumatic stress disorder, PTSD, Trauma‐ and stressor‐related disorders, Burnout, Behavioral health

## Abstract

**Objective:**

Burnout is widespread among behavioral health clinicians treating posttraumatic stress disorder (PTSD) among military populations. Intervention‐based strategies have shown some benefit in addressing clinician burnout. One Web‐based tool, the PTSD Clinicians Exchange, was designed to disseminate clinical best practices for the treatment of PTSD and facilitate self‐care to mitigate burnout. This study sought to determine whether this tool could reduce burnout among clinicians treating military populations.

**Methods:**

A total of 605 behavioral health clinicians from the U.S. Department of Veterans Affairs, the Department of Defense, and the community were enrolled in a randomized controlled trial to test the effectiveness of the PTSD Clinicians Exchange. Clinicians were assessed on demographic characteristics, practice patterns, and organizational support with an online survey at baseline and at 6 and 12 months. Burnout, secondary traumatic stress (STS), and compassion satisfaction were measured with the Professional Quality of Life Scale.

**Results:**

At baseline, no differences were observed in mean±SD burnout scores for the intervention (19.9±5.1) and control groups (20.2±5.4). Participation in the Exchange had no effect on burnout score at 12 months; burnout scores remained stable across the study period. In a multivariable stepwise regression model, older age, lower burnout at baseline, and lower STS scores and higher compassion satisfaction scores at 12 months were significantly associated with lower burnout scores.

**Conclusions:**

The PTSD Clinicians Exchange did not mitigate burnout among clinicians, possibly because of the content provided, the dissemination mechanism, or participants’ limited use of the Web site. These results can be used to inform and enhance future interventions.

## HIGHLIGHTS

- Burnout is a prevalent issue among behavioral health clinicians treating posttraumatic stress disorder (PTSD) in military populations.
- This study sought to determine whether a Web‐based tool could significantly reduce burnout among clinicians treating military populations that had experienced trauma.
- The PTSD Clinicians Exchange did not mitigate burnout among behavioral health clinicians.

Military populations exposed to combat are at risk for developing posttraumatic stress disorder (PTSD) (1, 2, 3). Behavioral health clinicians treating these populations face a number of challenges, including a high prevalence of PTSD among patients, increased caseloads, and long hours (4, 5, 6). Research has shown that these types of challenges can lead to burnout, a prevalent issue among the broader population of behavioral health clinicians (7). Estimates of burnout among behavioral health clinicians range from 21% to as high as 67% (8). Symptoms of burnout include feelings of hopelessness and difficulties in dealing with or doing work effectively (9). These symptoms have been found over time to degrade clinicians’ physical and mental well‐being, which can in turn affect clinicians’ personal lives (10), the quality of care provided (11), and the organizations in which they are employed (e.g., greater turnover and reduced productivity) (8, 12).

Given the consequences of clinician burnout, research has been conducted to evaluate factors that may decrease risk for the condition. Ballenger‐Browning and colleagues (4) found that having more confidants at work was associated with reduced risk of burnout. Additionally, greater transformational leadership, organizational support, and specialized clinician training are thought to be protective (13, 14, 15). Use of evidence‐based practices may also affect burnout by improving clinicians’ perceived self‐efficacy in treating PTSD (16); however, studies have found conflicting results (16, 17). Finally, compassion satisfaction, characterized by appreciation of the positive aspects of providing care, has also been found to protect against burnout (9, 18).

In addition to identifying protective factors, studies have evaluated the effectiveness of interventions designed to mitigate burnout (8, 19, 20, 21, 22). Most of these interventions consisted of trainings or workshops and have been found to have limited effectiveness over time (8, 19). Other studies focused on examining the utility of Web‐based interventions (21, 22) have reported mixed results. Given the range of effectiveness among interventions for burnout (19), research into more diverse approaches is needed. Thus, a Web‐based tool, the PTSD Clinicians Exchange, was designed to increase clinician familiarity with, perceived benefits of, and implementation of evidence‐based practices for the treatment of PTSD and to address clinician burnout. The Exchange built upon previous intervention efforts by using a Web‐based format and by linking clinicians with resources to enhance access to specialized training, which has been associated with reduced levels of burnout (15). Similar to previous interventions (22), the Exchange also provides clinicians with immediate feedback regarding their current level of burnout and suggests possible self‐care strategies to address burnout symptoms. Unlike previous efforts, the Exchange targets social support, an important protective factor against burnout (14, 23), by providing clinicians with the opportunity to connect with other colleagues and to receive feedback from experts. Given the number of protective factors offered to clinicians through use of the Exchange, this study sought to determine whether the Web site was effective in reducing clinician burnout. Secondary to this aim, levels of burnout across clinician settings were compared.

## Methods

### Intervention

The PTSD Clinicians Exchange is composed of three sections: “Engage,” which focuses on 26 key practices for PTSD; “Connect,” which includes a number of interactive features aimed at connecting clinicians with each other; and “Inspire,” a self‐care section (of primary interest in the present study) that provides resources aimed at managing stress, burnout, and secondary traumatic stress (STS). The Exchange also includes a self‐assessment component consisting of the Professional Quality of Life Scale–5 (ProQOL‐5) (9). By completing this self‐assessment, clinicians can ascertain their current level of burnout, STS, and compassion satisfaction.

### Sample

Data were collected as part of the PTSD provider registry study, a 12‐month randomized controlled trial of clinicians in the U.S. Department of Veterans Affairs (VA), Department of Defense (DOD), and general community beginning in May 2016, after approval by the institutional review boards of Stanford University and the New England Research Institutes. Of the 1,453 clinicians who responded to recruitment invitations sent via email and posted to online message boards for the behavioral health provider community, 792 consented to participate (55%). A total of 605 clinicians completed the baseline survey (76.4%) and were randomly assigned, with the use of a 3:1 randomization scheme, to either the intervention group with access to the Exchange or to the newsletter‐only control group. Participants in the intervention group were also sent biweekly email reminders featuring PTSD treatment practices included on the Web site. All participants were surveyed again at 6 and 12 months after baseline. The surveys consisted of questions to assess participants’ demographic characteristics, burnout status, and other covariates (e.g., years of experience treating veterans, compassion satisfaction, STS, practice patterns, and organizational support).

### Measures

Burnout, STS, and compassion satisfaction were measured with three 10‐item subscales within the ProQOL‐5 (9). This scale is the most commonly used measure of the positive and negative effects of working with individuals who have experienced traumatic events and has demonstrated exceptional construct validity (9, 14, 15, 16, 22, 24, 25, 26). Items were scored on a 5‐point scale (1, never, to 5, very often) and summed. Higher subscale scores indicate greater risk of burnout and STS or higher levels of compassion satisfaction. Cronbach's alpha ranged from 0.88 to 0.91, 0.81 to 0.84, and 0.82 to 0.84 across time points for the compassion satisfaction, burnout, and STS subscales, respectively.

To assess practice patterns, clinicians were asked about the total number of patients they treated during the past week and the number of those with PTSD. Clinicians also reported the number of hours over the past week spent on direct patient care and administrative activities and the total number of hours worked. The use of evidence‐based practices for PTSD was assessed by asking clinicians whether they had ever used prolonged exposure treatment, cognitive processing therapy, stress inoculation training, or eye movement desensitization and reprocessing.

Overall attitudes toward evidence‐based practices were measured using the Evidence‐Based Practice Attitudes Scale (EBPAS) (27). All fifteen items were rated on a 5‐point Likert scale (0, not at all, to 4, to a very great extent) and averaged (Cronbach's alpha over time points ranged from 0.82 to 0.84).

Organizational support was measured using an item developed for this study that assessed whether the clinician's primary supervisor was supportive of using “treatments supported by research.” This item was scored by using a 7‐point scale ranging from extremely unsupportive to extremely supportive; higher scores indicated greater support. An additional category of “NA, no primary supervisor” (not part of the 7‐point scale) was treated as the highest level of support for this analysis because of the lack of barriers to practice implementation when there is no supervisor. Specifically, a clinician who has no supervisor would be able to implement research‐supported treatments free of any supervisory barriers, as even a supportive supervisor may explicitly or implicitly limit which techniques the clinician uses.

Web site tracking metrics were integrated into the Exchange. Each participant in the intervention group was assigned a unique identifier to access the Web site, which was used to determine whether the participant used the Exchange. Participants’ Web site usage was categorized as having no access (control group), having access but never visiting the site, and having access and visiting the site once or more.

### Data Analysis

We completed all statistical analyses by using SAS, version 9.4, and we tested statistical significance at a level of 0.05. We used one maximum likelihood iteration (28) to impute missing data for the ProQOL‐5 and EBPAS scores, age, years of experience, number of clients, and hours, although these factors all had few missing data (range 2–18 missing items from 605 participants). Descriptive statistics were then calculated for all variables of interest. Associations between demographic characteristics and Exchange participation were examined by using chi‐square tests for categorical variables and t‐tests or Mann‐Whitney U tests for continuous variables. We used t‐tests to examine the associations between burnout and Exchange participation at baseline and 12 months. Because the primary outcome of interest was burnout scores at 12 months after baseline, we examined the associations between these scores and the variables of interest by using unadjusted linear regression models and then by using a multivariable analysis with backward elimination (step 1 below). We then constructed a stepwise linear regression model, with the first step based on the presence of significant predictors of burnout at 12 months found during the initial analysis above. Further steps were used to add a variety of clinical characteristics. In the last steps, the model examined group assignment, Web site usage, and use of evidence‐based practices.

## Results

### Sample Characteristics and Preintervention Equivalence

Characteristics of the participants are shown in Table 1. Most participants were Caucasian (75%), female (68%), and psychologists or social workers (76%), and participants had a mean age of 47. Forty‐three percent of participants were from the VA, 18% were from the DOD, and 39% were from the community. Participants reported an average of 24 clients per week, with 11 of those having a diagnosis of PTSD. The clinicians also reported working an average of 40 hours per week, with 25 of those hours spent on direct client care and 12 on administrative activities. Most of the sample (84%) reported using at least one of the four evidence‐based practices for PTSD listed on the Exchange. No significant differences were found among the intervention and control groups for demographic or other baseline measures. Among the 605 participants, 379 (63%) completed the assessment at 6 months, 395 (65%) completed the assessment at 12 months, and 311 subjects (51%) completed both assessments. Because completion of assessments varied by practice setting, we controlled for setting in all analyses.

**TABLE 1:** Baseline demographic characteristics for 605 behavioral health clinicians, by PTSD Clinicians Exchange intervention group and control group

	Intervention group (N=453)		Control group (N=152)	
Characteristic	M	SD	M	SD
Age	48.3	11.4	46.5	12.2
Years treating mental illness	17.5	10.1	16.2	10.0
Years treating veterans	8.8	7.0	8.0	6.1
Number of clients per week	23.5	12.3	25.4	12.6
Number of clients with posttraumatic stress disorder	11.2	11.4	11.6	12.0
Hours per week of administrative work	12.3	7.6	12.2	7.3
Hours per week of client care	24.3	9.1	24.2	8.9
Organizational support^a^	6.2	1.4	6.2	1.2
Compassion satisfaction score^b^	42.6	4.9	42.5	4.9
Burnout score^b^	19.9	5.1	20.2	5.4
Secondary traumatic stress score^b^	18.9	4.9	19.0	5.2

	Median	Range	Median	Range
Evidence‐Based Practice Attitudes Scale score^c^	4.0	2.1–5.0	4.0	2.8–5.0

	**N**	**%**	**N**	**%**
Practice setting				
Veterans Affairs	197	44	66	43
Department of Defense	81	18	27	18
Community	175	39	59	39
Discipline				
Social worker	187	41	58	38
Psychologist	157	35	60	40
Professional mental health counselor	85	19	28	18
Medical professional with psychiatry focus	16	4	5	3
Other/missing	8	2	1	1
Gender				
Female	308	68	110	72
Male	140	32	41	27
Other	5	1	1	1
Race‐ethnicity				
Caucasian	339	75	122	80
African American	34	8	13	9
Hispanic	21	5	4	3
Asian	14	3	3	2
Mixed	17	4	8	5
Other/missing	28	6	2	1

^a^ Organizational support refers to support for using evidence‐based practices.

^b^ Compassion satisfaction score, burnout score, and secondary traumatic stress score were measured with three 10‐item subscales within the Professional Quality of Life Scale–5. Possible scores range from 10 to 50, with higher scores indicating greater risk of burnout.

^c^ Possible scores on the Evidence‐Based Practice Attitudes Scale range from 1 to 5, with higher scores indicating more favorable provider attitudes toward adopting evidence‐based practices.

### Relationship Between Burnout and Study Variables

**FIGURE 1:**
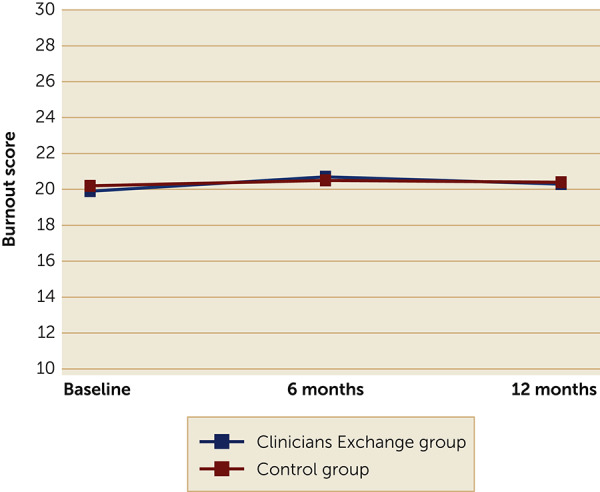
ProQOL‐5 burnout score over time among 605 behavioral health clinicians enrolled and not enrolled in PTSD Clinicians Exchange, by randomization group assignment^a^ ^a^ProQOL‐5, Professional Quality of Life Scale–5. Possible scores range from 10 to 50, with higher scores indicating greater risk of burnout. At baseline, p=0.81, at 6 months p=0.85, at 12 months, p=0.96. All p values are nonsignificant.

For the primary outcome of interest, burnout scores over time, we found no significant differences between treatment arms (Figure 1), even when we controlled for other factors in the regression analyses. At baseline, mean±SD scores of burnout for the intervention and control groups, respectively, were 19.9±5.1 and 20.2±5.4; at 6 months, mean burnout scores were 20.7±5.5 and 20.5±5.4; and at 12 months mean scores were 20.3±5.4 and 20.4±5.6.

The results from our unadjusted regression models (Table 2) showed that greater age (*β*=–0.28, p<0.001), more experience treating mental illness (*β*=–0.17, p<0.01), more organizational support for using treatment supported by research (*β*=–0.24, p<0.001), and a higher score on compassion satisfaction at 12 months (*β*=–0.75, p<0.001) were inversely related to 12‐month burnout scores. On the other hand, having a larger caseload of clients with PTSD (*β*=0.21, p<0.001), more administrative hours (*β*=0.12, p<0.05), a higher baseline burnout score (*β*=0.75, p<0.001), and a higher STS score at 12 months (*β*=0.56, p<0.001) were predictive of having significantly higher 12‐month burnout scores. Clinicians in the DOD (*β*=–0.16, p<0.01) and community (*β*=–0.34, p<0.001) sectors had significantly lower burnout scores compared with participants in the VA.

**TABLE 2:** Predictors of burnout at 12 months among 605 behavioral health clinicians enrolled and not enrolled in PTSD Clinicians Exchange

Characteristic	*β*	SE	95% CI
Age	–0.28***	0.02	–0.09, –0.17
Years treating mental illness	–0.17**	0.03	–0.04, –0.16
Years treating veterans	–0.08	0.04	0.01, –0.15
Number of clients	–0.03	0.02	0.03, –0.05
Number of clients with posttraumatic stress disorder	0.21***	0.02	0.14, 0.06
Hours of administrative work per week	0.12*	0.04	0.17, 0.01
Hours of client care per week	–0.05	0.03	0.03, –0.09
Organizational support^a^	–0.24***	0.19	–0.52, –1.26
Burnout score at baseline^b^	0.75***	0.04	0.86, 0.70
Compassion satisfaction score at 12 months^b^	–0.75***	0.03	–0.68, –0.80
Secondary traumatic stress score at 12 months^b^	0.56***	0.04	0.65, 0.49
Evidence‐Based Practice Attitudes Scale score^c^	–0.06	0.57	0.50, −1.74
Use of evidence‐based practices	–0.05	0.77	0.83, −2.19
Practice setting (reference: Veterans Affairs)			
Department of Defense	–0.16**	0.78	–0.90, –3.96
Community	–0.34***	0.58	–2.73, –5.01
Discipline (reference: social worker)			
Psychologist	0.03	0.64	1.61, –0.89
Professional mental health counselor	–0.11	0.76	0.02, −2.96
Medical professional with psychiatry focus	–0.03	1.86	2.58, −4.72
Other/missing	0.01	2.47	5.53, −4.15
Gender (reference: female)			
Male	0.04	0.62	1.74, –0.70
Other/missing gender	–0.03	2.46	3.42, −6.22
Race‐ethnicity (reference: Caucasian)			
African American	–0.01	1.09	1.86, −2.42
Hispanic	–0.06	1.40	1.05, −4.43
Asian	0.08	1.95	7.00, −0.64
Mixed	0.00	1.54	2.96, −3.08
Other/missing	–0.07	1.26	2.31, −2.63

^a^ Organizational support refers to support for using evidence‐based practices.

^b^ Burnout score, compassion satisfaction score, and secondary traumatic stress score were measured with three 10‐item subscales within the Professional Quality of Life Scale–5. Possible scores range from 10 to 50, with higher scores indicating greater risk of burnout.

^c^ Possible scores on the Evidence‐Based Practice Attitudes Scale range from 1 to 5, with higher scores indicating more favorable provider attitudes toward adopting evidence‐based practices.

*p<0.05, **p<0.01, ***p<0.001.

A stepwise linear regression model was developed to further examine the relationship between participant characteristics and treatment group assignment with burnout. As shown in Table 3, baseline burnout score, 12‐month compassion satisfaction score, 12‐month STS score, and age were entered in step 1. Each of the variables entered in step 1 were significant predictors of burnout score at 12 months. Practice setting, years of experience treating mental illness, hours spent on administrative work, organizational support for using evidence‐based practices, number of clients with PTSD, and EBPAS scores were entered in steps 2–7, respectively. None of these variables were found to significantly predict burnout score. We entered treatment arm in step 8 and use of evidence‐based practice in step 9, neither of which significantly predicted burnout at 12 months (Table 3, Model 1). Because only a third of those randomized to the intervention group accessed the Web site, Web site usage was entered in the model (Table 3, Model 2) to further investigate its effect on burnout. Similar to treatment group assignment, Web site usage was not associated with burnout at 12 months.

**TABLE 3:** Predictors of burnout at 12 months among 605 behavioral health clinicians enrolled and not enrolled in PTSD Clinicians Exchange, by randomization group (model 1) and Web site usage (model 2)

Step	Characteristic	Model 1: randomization group				Model 2: Web site usage			
		R^2^	β	SE	95% CI	R^2^	β	SE	95% CI
1	Burnout score at baseline^a^	0.7588	0.33***	0.04	0.42, 0.26	0.7588	0.33***	0.04	0.42, 0.26
	Compassion satisfaction score at 12 months^a^		−0.44***	0.03	−0.37, −0.49		−0.44***	0.03	−0.37, −0.49
	Secondary traumatic stress score at 12 months^a^		0.26***	0.03	0.33, 0.21		0.27***	0.03	0.33, 0.21
	Age		−0.09*	0.02	0.00, −0.08		−0.09*	0.02	0.00, −0.08
2	Practice setting	0.7654				0.7654			
	Department of Defense vs. Veterans Affairs		0.01	0.45	0.99, −0.77		0.01	0.45	1.03, −0.73
	Community vs. Veterans Affairs		−0.04	0.43	0.36, −1.32		−0.05	0.44	0.26, −1.46
3	Years treating mental illness	0.7658	0.02	0.02	0.05, −0.03	0.7658	0.02	0.02	0.05, −0.03
4	Hours of administrative work per week	0.7669	0.04	0.02	0.07, −0.01	0.7669	0.04	0.02	0.07, −0.01
5	Organizational support^b^	0.7692	−0.04	0.11	0.07, −0.37	0.7692	−0.04	0.11	0.09, −0.35
6	Number of clients with posttraumatic stress disorder	0.7693	0.01	0.02	0.04, −0.04	0.7693	0.01	0.02	0.04, −0.04
7	Evidence‐Based Practice Attitudes Scale score^c^	0.7714	0.02	0.31	0.85, −0.37	0.7714	0.02	0.31	0.86, −0.36
8	Control vs. Exchange	0.7716	−0.01	0.32	0.48, −0.78	—	—	—	
	Control vs. Exchange use	—	—	—	—	0.7736	0.02	0.38	1.00, −0.48
	No use vs. Exchange use	—	—	—	—		0.06	0.35	1.36, −0.02
9	Use of evidence‐based practices	0.7738	0.05	0.41	1.56, −0.04	0.7762	0.05	0.41	1.63, 0.03

^a^ Burnout, compassion satisfaction, and secondary traumatic stress were measured with three 10‐item subscales within the Professional Quality of Life Scale–5.

^b^ Organizational support refers to support for using evidence‐based practices.

^c^ Possible scores on the Evidence‐Based Practice Attitudes Scale range from 1 to 5, with higher scores indicating more favorable provider attitudes toward adopting evidence‐based practices.

*p<0.05, ***p<0.001.

## Discussion

The PTSD Clinicians Exchange, an innovative Web resource, was developed with the primary goal of increasing behavioral health clinicians’ familiarity with evidence‐based practices for the treatment of PTSD and increasing the perceived benefits and implementation of these practices. In addition to providing treatment resources, the Web site was designed to enable clinicians to connect with one another via the Clinicians’ Corner and to provide self‐care resources, including ways to self‐assess for burnout. While reducing clinician burnout was not the main goal of the Exchange, several features target a number of protective factors that could mitigate burnout (4, 16). Although it was anticipated that clinicians’ main focus in using the Web site would be to identify resources for clinical best practices for the treatment of PTSD, we hypothesized that the use of the additional self‐care resources would reduce burnout. However, Exchange participation did not significantly reduce burnout during the study, even after accounting for clinician use (some or none) of the Web site.

Although the Exchange uses a unique approach to address burnout, previous burnout interventions have had varying levels of success, so these results were not completely unexpected. The Web‐based self‐care program developed by Shoji et al. (21), whose only aim was to reduce burnout and STS symptoms, did not significantly reduce burnout for the Web‐only group, nor did an online intervention designed for physicians (29). A mobile app for reducing burnout designed by Wood et al. (22), however, was found to significantly reduce burnout, suggesting that mobile technologies may be more effective for motivating busy clinicians to access self‐care resources, particularly during nonwork hours. Adapting our tool so these resources could be used at the times and places chosen by the clinicians via an app may have led to greater use of the Web site; only a third of the clinicians in the intervention group accessed the Exchange. Although baseline burnout was not found to be associated with use of the Web site, in a study of how the Exchange was used by participants, we found that lower burnout was predictive of more visits to the Web site and more pages viewed, indicating that burnout may have played a role in how the clinicians interacted with the Web site. Furthermore, as compared with national norms (9), we may not have seen an effect of the Web site because of the high average level of burnout among clinicians in our sample.

Although the intervention did not reduce burnout, we identified baseline predictors of burnout at 12 months that may help optimize future interventions. Our results were fairly consistent with previous research: younger age, less experience and organizational support, lower compassion satisfaction score, higher baseline burnout and STS scores, increased caseloads of patients with PTSD, and more administrative hours were all associated with higher burnout scores (4, 6, 9, 13, 14, 24, 25, 30). The findings also suggested differences in levels of burnout across service sectors, with VA clinicians reporting significantly higher burnout scores compared with DOD and community clinicians. This finding is surprising given that the VA has a number of targeted self‐care resources designed to reduce burnout, which are less available among community clinicians. However, other organizational factors, which are less prevalent in other sectors, may predispose VA clinicians to burnout (e.g., less diagnostic diversity in their caseloads and more administrative burden on clinicians) (31). We also found that use of evidence‐based practices did not reduce burnout, a finding that was somewhat consistent with previous equivocal findings (16, 17). Additionally, these null findings may have been due to the large percentage of clinicians in our sample who reported already using evidence‐based practices to treat patients with PTSD.

The results from our multivariable models, showing that the only significant predictors of 12‐month burnout across all steps were baseline burnout, 12‐month STS score, 12‐month compassion satisfaction score, and age, suggest that baseline burnout and other factors may contribute to the maintenance of burnout over time. Compassion satisfaction at 12 months was also shown to have the largest effect size on burnout score at 12 months. This finding confirmed previous research, which found that increased compassion satisfaction scores are associated with reduced burnout, because work is seen as a source of personal gratification rather than a source of fatigue or emotional exhaustion (9, 18).

One noteworthy limitation to this study is the low number of participants in the intervention group who accessed the Exchange. While this finding is typical of Web site engagement when no incentive to participate is offered, it limited our ability to assess whether Exchange features effectively reduced burnout. However, this pattern of Web site engagement is likely to reflect how clinicians would use this tool in the real world. Also, while participants were randomly selected for the Exchange group or the newsletter‐only control group, the overall sample consisted of self‐selected volunteers, which may have introduced unmeasured response biases. Additionally, self‐care resources were a relatively small part of the Exchange overall.

## Conclusions

In this study, we found average levels of burnout across clinicians in the VA, DOD, and community sectors treating military populations that had experienced trauma, and we found significantly higher levels of burnout among clinicians in the VA compared with those in the DOD and community sectors. Although the PTSD Clinicians Exchange did not reduce burnout, it may be a valuable tool for clinicians looking for resources to improve their practice. One possible explanation for our findings is that the self‐care content provided on the Web site was not perceived as relevant for mitigating burnout. Instead, clinicians may perceive content promoting organizational support or work satisfaction as more useful. The results were striking in how few clinicians accessed the Web site. Given burnout's far‐reaching effects, from implications for the individual patient to the organization as a whole, organizations should explore ways to engage clinicians in targeted burnout interventions and provide resources to promote self‐care (32). Findings from the present study indicate that a Web‐based dissemination tool focused on self‐care is unlikely to be used by those who need it most. Busy clinicians who are at risk for burnout are unlikely to add additional tasks to their to‐do list. Although it was not tested in this study, a different dissemination method, such as an app that can be accessed in a variety of settings (22), may be more likely to be used by busy clinicians. Future research should build on these findings by investigating the relative importance of dissemination methods versus intervention content for reducing burnout.

## References

[1] Kok BC , Herrell RK , Thomas JL , et al: Posttraumatic stress disorder associated with combat service in Iraq or Afghanistan: reconciling prevalence differences between studies. J Nerv Ment Dis 2012; 200:444–450 22551799 10.1097/NMD.0b013e3182532312

[2] Kang HK , Natelson BH , Mahan CM , et al: Post‐traumatic stress disorder and chronic fatigue syndrome‐like illness among Gulf War veterans: a population‐based survey of 30,000 veterans. Am J Epidemiol 2003; 157:141–148 12522021 10.1093/aje/kwf187

[3] Kulka RA , Schlenger WE , Fairbank JA , et al: Trauma and the Vietnam War Generation: Report of Findings From the National Vietnam Veterans Readjustment Study. New York, Brunner/Mazel, 1990

[4] Ballenger‐Browning KK , Schmitz KJ , Rothacker JA , et al: Predictors of burnout among military mental health providers. Mil Med 2011; 176:253–260 21456349 10.7205/milmed-d-10-00269

[5] Linnerooth PJ , Mrdjenovich AJ , Moore BA : Professional burnout in clinical military psychologists: recommendations before, during, and after deployment. Prof Psychol Res Pr 2011; 42:87–93

[6] Kok BC , Herrell RK , Grossman SH , et al: Prevalence of professional burnout among military mental health service providers. Psychiatr Serv 2016; 67:137–140 26567929 10.1176/appi.ps.201400430

[7] Maslach C , Schaufeli WB , Leiter MP : Job burnout. Annu Rev Psychol 2001; 52:397–422 11148311 10.1146/annurev.psych.52.1.397

[8] Morse G , Salyers MP , Rollins AL , et al: Burnout in mental health services: a review of the problem and its remediation. Adm Policy Ment Health Ment Health Serv Res 2012; 39:341–352 10.1007/s10488-011-0352-1 PMC3156844 21533847

[9] Stamm BH : The Concise ProQOL Manual, 2nd ed. Pocatello, ID, ProQOL.org, 2010

[10] Jackson SE , Maslach C : After‐effects of job‐related stress: families as victims. J Organ Behav 1982; 3:63–77

[11] Leiter MP , Harvie P , Frizzell C : The correspondence of patient satisfaction and nurse burnout. Soc Sci Med 1998; 47:1611–1617 9823056 10.1016/s0277-9536(98)00207-x

[12] Cocker F , Joss N : Compassion fatigue among healthcare, emergency and community service workers: a systematic review. Int J Environ Res Public Health 2016; 13:618 10.3390/ijerph13060618 PMC4924075 27338436

[13] Green AE , Albanese BJ , Shapiro NM , et al: The roles of individual and organizational factors in burnout among community‐based mental health service providers. Psychol Serv 2014; 11:41–49 24564442 10.1037/a0035299 PMC4294456

[14] Handran J : Trauma‐informed systems of care: the role of organizational culture in the development of burnout, secondary traumatic stress, and compassion satisfaction. Journal of Social Welfare and Human Rights 2015; 3:1–22

[15] Sprang G , Clark JJ , Whitt‐Woosley A : Compassion fatigue, compassion satisfaction, and burnout: factors impacting a professional's quality of life. J Loss Trauma 2007; 12:259–280

[16] Craig CD , Sprang G : Compassion satisfaction, compassion fatigue, and burnout in a national sample of trauma treatment therapists. Anxiety Stress Coping 2010; 23:319–339 19590994 10.1080/10615800903085818

[17] Garcia HA , McGeary CA , Finley EP , et al: Evidence‐based treatments for PTSD and VHA provider burnout: the impact of cognitive processing and prolonged exposure therapies. Traumatology 2015; 21:7–13

[18] Wagaman MA , Geiger JM , Shockley C , et al: The role of empathy in burnout, compassion satisfaction, and secondary traumatic stress among social workers. Soc Work 2015; 60:201–209 26173361 10.1093/sw/swv014

[19] Awa WL , Plaumann M , Walter U : Burnout prevention: a review of intervention programs. Patient Educ Couns 2010; 78:184–190 19467822 10.1016/j.pec.2009.04.008

[20] Program helps bolster ‘resilience’ of military healthcare providers. US Army Europe Public Affairs Office, December 2, 2008. https://www.army.mil/article/14683/program_helps_bolster_resilience_of_military_health_care_providers. Accessed March 16, 2019.

[21] Shoji K , Benight CC , Stearns S : SupportNet: a randomized controlled trial for military behavioral health burnout, in Secondary Trauma and Burnout in Military Behavioral Health Providers. Edited by Benight CC . New York, Palgrave Macmillan, 2016

[22] Wood AE , Prins A , Bush NE , et al: Reduction of burnout in mental health care providers using the provider resilience mobile application. Community Ment Health J 2017; 53:452–459 28070775 10.1007/s10597-016-0076-5

[23] Ortlepp K , Friedman M : Prevalence and correlates of secondary traumatic stress in workplace lay trauma counselors. J Trauma Stress 2002; 15:213–222 12092913 10.1023/A:1015203327767

[24] Cieslak R , Shoji K , Douglas A , et al: A meta‐analysis of the relationship between job burnout and secondary traumatic stress among workers with indirect exposure to trauma. Psychol Serv 2014; 11:75–86 23937082 10.1037/a0033798

[25] Deighton RM , Gurris N , Traue H : Factors affecting burnout and compassion fatigue in psychotherapists treating torture survivors: is the therapist's attitude to working through trauma relevant? J Trauma Stress 2007; 20:63–75 17345651 10.1002/jts.20180

[26] Alkema K , Linton JM , Davies R : A study of the relationship between self‐care, compassion satisfaction, compassion fatigue, and burnout among hospice professionals. J Soc Work End Life Palliat Care 2008; 4:101–119 19042895 10.1080/15524250802353934

[27] Aarons GA : Mental health provider attitudes toward adoption of evidence‐based practice: the Evidence‐Based Practice Attitude Scale (EBPAS). Ment Health Serv Res 2004; 6:61–74 15224451 10.1023/b:mhsr.0000024351.12294.65 PMC1564126

[28] Graham JW : Missing Data: Analysis and Design. New York, Springer, 2012

[29] Dyrbye LN , West CP , Richards ML , et al: A randomized, controlled study of an online intervention to promote job satisfaction and well‐being among physicians. Burn Res 2016; 3:69–75

[30] Leiter MP , Harvie PL : Burnout among mental health workers: a review and a research agenda. Int J Soc Psychiatry 1996; 42:90–101 8811393 10.1177/002076409604200203

[31] Voss Horrell SC , Holohan DR , Didion LM , et al: Treating traumatized OEF/OIF veterans: how does trauma treatment affect the clinician? Prof Psychol Res Pr 2011; 42:79–86

[32] Shanafelt TD , Noseworthy JH : Executive leadership and physician well‐being: nine organizational strategies to promote engagement and reduce burnout. Mayo Clin Proc 2017; 92:129–146 27871627 10.1016/j.mayocp.2016.10.004

